# Extreme weather event in spring 2013 delayed breeding time of Great Tit and Blue Tit

**DOI:** 10.1007/s00484-014-0816-6

**Published:** 2014-03-23

**Authors:** Michał Glądalski, Mirosława Bańbura, Adam Kaliński, Marcin Markowski, Joanna Skwarska, Jarosław Wawrzyniak, Piotr Zieliński, Jerzy Bańbura

**Affiliations:** 1 Department of Experimental Zoology and Evolutionary Biology, Faculty of Biology and Environmental Protection, University of Łódź, Banacha 12/16, 90-237 Łódź, Poland; 2 Museum of Natural History, Faculty of Biology and Environmental Protection, University of Łódź, Kilińskiego 101, 90-011 Łódź, Poland; 3 Department of Teacher Training and Biological Diversity Studies, Faculty of Biology and Environmental Protection, University of Łódź, Banacha 1/3, 90-237 Łodź, Poland; 4 Department of Ecology and Vertebrate Zoology, Faculty of Biology and Environmental Protection, University of Łódź, Banacha 12/16, 90-237 Łódź, Poland

**Keywords:** *Cyanistes caeruleus*, Blue Tit, *Parus major*, Great Tit, Climate change, Laying date

## Abstract

The impact of climatic changes on life cycles by re-scheduling the timing of reproduction is an important topic in studies of biodiversity. Global warming causes and will probably cause in the future not only raising temperatures but also an increasing frequency of extreme weather events. In 2013, the winter in central and north Europe ended late, with low temperatures and long-retained snow cover—this extreme weather phenomenon acted in opposition to the increasing temperature trend. In 2013, thermal conditions measured by the warmth sum in the period 15 March–15 April, a critical time for early breeding passerines, went far beyond the range of the warmth sums for at least 40 preceding years. Regardless of what was the reason for the extreme early spring 2013 and assuming that there is a potential for more atypical years because of climate change, we should look closely at every extreme phenomenon and its consequences for the phenology of organisms. In this paper, we report that the prolonged occurrence of winter conditions during the time that is crucial for Blue Tit (*Cyanistes caeruleus*) and Great Tit (*Parus major*) reproduction caused a substantial delay in the onset of egg laying in comparison with typical springs.

## Introduction

The dynamics of bird populations are influenced by climate change on both regional and local scales (Crick and Sparks 1999; Hallett et al. 2004; Crick 2004; Robinson et al. 2007; Fletcher et al. 2013). There is good evidence from Europe and North America that many avian species nowadays migrate and breed earlier as a result of higher temperatures (Crick 1997; Tryjanowski et al. 2002; Huppop and Huppop 2003; Parmesan 2007; Bauer et al. 2010; Matthysen et al. 2010; Chmielewski et al. 2013). The impact of the changes varies for different latitude, longitude, topography, etc., as well as among different species (Sanz 2002; Askeyev et al. 2010; Goodenough et al. 2010, 2011). Yet, global warming not only means higher temperatures but also more frequent extreme weather events (Ogi et al. 2010; Wu et al. 2012; Zhang et al. 2012; Coumou and Rahmstorf 2012; Tang et al. 2013). In light of many recent extreme events all over the globe in 2012 and 2013 (Peterson et al. 2013; Vaughan et al. 2013; Zscheischler et al. 2013; some of which are considered to be the result of climate change), there is a possibility that distinctly low temperatures in the early spring 2013 could probably be linked to climate change. On the other hand, some climatologists think that there is no link between recent extreme events and climate change (Wallace et al. 2014), but regardless of what is the reason for the extreme spring 2013 at our area and assuming that there is a potential for more frequent occurrence of atypical years, in the course of climate changes, we should study extreme weather phenomena and their consequences for wild populations.

In 2013, the winter ended late and with very low temperatures in central and north Europe. March 2013 was the coldest in the UK meteorological records since 1962 (measurement since 1910; Slingo 2013), and in central Europe, the temperatures were also much below recent averages, and as a result, many temperature records were beaten (Andrews 2013; Harris 2013). Because of unusually low late winter/early spring temperatures, for many European countries, March 2013 was the snowiest March in at least 400 years (Harris 2013). In central Poland, snow cover was still almost complete until 9 April in urban parks and until 15 April in forests (Glądalski, personal observation). According to some researchers, the basic cause of those extreme weather phenomena could be the melting of arctic ice cap, which caused a long-lasting inflow of cold air to Europe (Li et al. 2012; Ravilious 2013; Slingo 2013). Some authors think that there may be existing connection between arctic sea ice loss caused by global warming and large-scale circulation patterns in the Northern Hemisphere (Francis et al. 2009; Francis and Vavrus 2012; Jaiser et al. 2012; Screen et al. 2013; Tang et al. 2013).

Fletcher et al. (2013) conclude that there is a need to collect long-term phenology monitoring data in order to fully understand the impacts of climate change on different species. Bauer et al. (2010) note in addition that, surprisingly, most papers analysing these trends do not use data from central Europe, and there is a need to fill this gap. The aim of this paper is to show the influence of an extreme weather event in spring 2013 (unique over at least 40 years) on the timing of breeding of Great Tits (*Parus major*) and Blue Tits (*Cyanistes caeruleus*) by comparison with the data from the preceding decade.

## Materials and methods

This study was carried out in 1999–2013 as part of a long-term project of research into the breeding biology of hole-nesting birds occupying nest boxes near Łódź (51° 47′ N, 19° 28′ E, central Poland). Study areas are located in two, 10-km-distant, structurally and floristically contrasting types of habitats: an urban parkland and a rich deciduous forest. Data analysed include tit breeding seasons (April–May) 1999–2013 in the parkland and 2002–2013 in the forest (Marciniak et al. 2007; Kaliński et al. 2012). Both areas have been equipped with wooden nest boxes (Labmrechts et al. 2010), c. 200 in the parkland area and c. 300 in the forest. In spring, all nest boxes were checked for first egg dates and other breeding characteristics of tits. A total of 1,182 first clutches of Great Tit (735 from the parkland and 447 from the forest) and 861 first clutches of Blue Tit (412 from parkland and 449 from the forest) were studied between 1999 and 2013.

The local temperatures for Łódź were obtained from TuTiempo.net climate database (http://www.tutiempo.net/en/Climate/LODZ/124650.htm). Following Perrins and McCleery (1989), we calculated warmth sums, as the sum of the maximum daily temperatures for the period between 15 March and 15 April each year, to characterise thermal conditions. We used Cook’s distance to measure how extreme 2013 was in comparison with the trend for 14 preceding years (being the background for our phenological data on tits). In addition, in order to compare the 15 March–15 April warmth sum for 2013 with the warmth sums for a longer period encompassing 40 years of available records (1973–2012), we used a special version of *t* test described by Sokal and Rohlf (1994). We used one-way ANOVA followed by Dunnett’s post hoc test to compare mean laying dates between years.

Graphical and statistical analyses were conducted applying STATISTICA 6 (StatSoft, Inc., 2003).

## Results

**Fig. 1:**
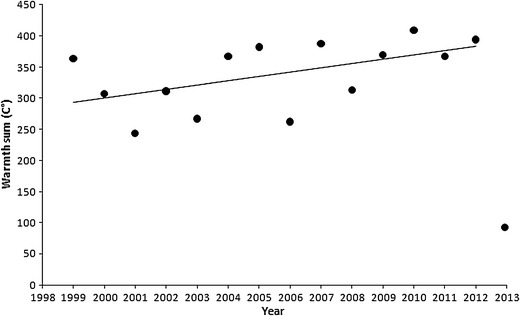
Warmth sums (sums of the daily maximum temperatures between 15 March and 15 April) by year for the period 1999–2013, including temporal trend line (excluding 2013)

The warmth sum was extremely low in 2013 in comparison with the values for the preceding 14 years (Fig. 1). When we excluded 2013, there was a trend for warmth sums to increase during 1999–2012 (*r* = 0.53, *n* = 14, *p* = 0.049) (Fig. 1). The mean warmth sum for the period 1999–2012 was 338.5 °C, increasing by 2.34 °C per year (the mean for 2002–2012 was 347.8 °C), compared to 90.2 °C in 2013. The Cook’s distance for the 2013 data point in temporal trend line shows that this point is a very strong outlier (4.62), whereas the median distance (1999–2013) is 0.03, while the second highest distance in the analysed period is only 0.14 (2012) (Fig. 1). When the data point for 2013 is included into the regression, the relationship between the warmth sum and years becomes nonsignificantly negative. The 15 March–15 April warmth sum for 2013 is extremely low also in a wider comparison with the warmth sums for the preceding 40 years. The value for 2013 is below the lowest marginal warmth sum in 1973–2012 (ranging from 185.5 to 436 °C; mean 315.1 °C ± 64.3 SD), resulting in a highly significant difference (*t* = 3.46, *df* = 39, *p* < 0.001).

The yearly mean date of first egg laying was highly negatively correlated with the warmth sums over the study years at both study sites for both tit species: for Great Tits, *r* = −0.79, *n* = 15, *p* < 0.001 in the parkland (1999–2013) and *r* = −0.81, *n* = 12, *p* = 0.002 in the forest (2002–2013), and for Blue Tits, *r* = −0.77, *n* = 15, *p* < 0.001 in the parkland (1999–2013) and *r* = −0.85, *n* = 12, *p* < 0.001 in the forest (2002–2013).

**Fig. 2:**
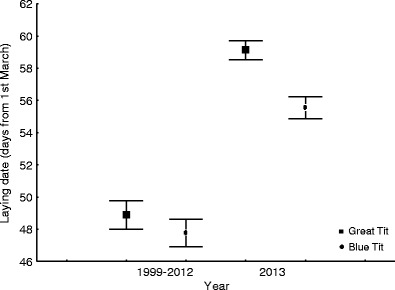
Mean laying dates (days from 1 March) of Great Tits and Blue Tits in 1999–2012 vs. 2013 in the parkland site. Mean laying dates are presented as averages ± 95 % standard error intervals

**Fig. 3:**
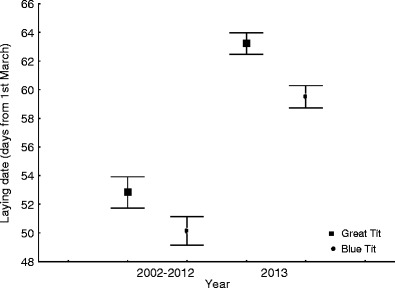
Mean laying dates (days from 1 March) of Great Tits and Blue Tits in 2002–2012 vs. 2013 in the forest site. Mean laying dates are presented as averages ± 95 % standard error intervals

At both study sites, the mean laying dates each year in the period 1999–2012 (parkland) and 2002–2012 (forest) are earlier than in 2013 (ANOVA: for the Great Tit in the parkland, *F*_14,721_ = 38.95, *p* < 0.001, and in the woodland, *F*_11,436_ = 48.20, *p* < 0.001, and for the Blue Tit in the parkland, *F*_14,397_ = 19.48, *p* < 0.001, and in the woodland, *F*_11,437_ = 44.09, *p* < 0.001) (Figs. 2 and 3). All differences between 2013 and the preceding years were significant for both species, except for Blue Tits in parklands in 2003 and 2006 (post hoc Dunnett’s tests) (*p* < 0.01; data not shown).

## Discussion

Mean dates of first egg laying by Great Tits and Blue Tits were highly correlated with spring (pre-laying) thermal conditions (in our region, the period between 15 March and 15 April is crucial; Glądalski 2013), with earlier laying occurring in warmer years. The temperature trend (1999–2012), being the background for our phenological data, shows gradual warming. Yet, the 15 March and 15 April warmth sum in 2013 was lower than that in any spring during 1973–2012. In 2013, the beginning of spring was extremely cold, and therefore, initiation of breeding was significantly delayed. Some delays in the timing of breeding were also found for both tit species in 2003 and 2006. Although the spring warmth sums in 2003 and 2006 were not distinctly low, the preceding winters were relatively severe, with the long-lasting snow cover and some adverse weather spells occurring even in early April, which could prevent females from early laying (Glądalski 2013).

Currently, many avian species in Europe migrate and breed earlier as a result of higher temperatures caused by global climate changes (Crick and Sparks 1999; Tryjanowski et al. 2002; Both et al. 2004; Both and Visser 2005; Parmesan 2007; Matthysen et al. 2010; Fletcher et al. 2013). The Great Tit and the Blue Tit are among such earlier breeding species (Potti 2009; Bauer et al. 2010; Smallegange et al. 2010; Chmielewski et al. 2013). However, the force of impact of climate warming on reproductive parameters of both tit species seems to vary depending on variables such as longitude, latitude, topography, orientation of slopes, etc., and as a result, in some areas, it may be too weak to be detectable (Sanz 2002). The effect is stronger for the populations of birds in western Europe (including Mediterranean area), where warmer and wetter winters in recent times result in accelerated breeding. The effect in eastern Europe is weaker—it has a less distinct influence on fewer species (Sanz 2002; Visser et al. 2003; Zalakevicius et al. 2005; Gordo et al. 2011).

Our study shows that extreme phenomena may act in opposition to trends, but more data are needed, as 1 year is certainly not enough to draw too far-reaching conclusions. Therefore, extreme weather phenomena that influence wild avian populations should be recorded, especially because there is a potential for more frequent occurrence of atypical years associated with climate change. It was extensively debated whether or not different species would be able to adapt quickly enough to keep up with gradually changing environment (Visser 2008), but little attention was devoted to potential consequences of extreme weather events occurring more often. The frequency of extremes in weather will probably increase (Zhang et al. 2012), and it will have consequences for life histories of different organisms (Robinson et al. 2007). Probably more extreme weather events and strong deviations from long-term trends, being very strong but unpredictable factors, could disturb life history strategies of some species and make it more difficult for organisms to adapt to local environments (Visser 2008; Chamberlain and Pearce-Higgins 2013; Fletcher et al. 2013). Maybe this one outlier does not make a wider story by itself, but if similar outliers will appear more frequently, as there is potential for more atypical years as the result of climate change, then further investigations should focus on the impact of extreme events on different life history traits, such as clutch size, hatching rate, reproductive success and proportions of second clutches.
